# Complete and Draft Genome Sequences of 12 Plant-Associated *Rathayibacter* Strains of Known and Putative New Species

**DOI:** 10.1128/MRA.00316-20

**Published:** 2020-05-28

**Authors:** Sergey V. Tarlachkov, Irina P. Starodumova, Lubov V. Dorofeeva, Natalia V. Prisyazhnaya, Semen A. Leyn, Jaime E. Zlamal, Marinela L. Elane, Andrei L. Osterman, Steven A. Nadler, Sergei A. Subbotin, Lyudmila I. Evtushenko

**Affiliations:** a All-Russian Collection of Microorganisms (VKM), G. K. Skryabin Institute of Biochemistry and Physiology of Microorganisms, Pushchino Scientific Center for Biological Research of the Russian Academy of Sciences, Pushchino, Russia; b Branch of Shemyakin and Ovchinnikov Institute of Bioorganic Chemistry of the Russian Academy of Sciences, Pushchino, Russia; c Infectious and Inflammatory Disease Center, Sanford Burnham Prebys Medical Discovery Institute, La Jolla, California, USA; d Department of Entomology and Nematology, University of California, Davis, California, USA; e California Department of Food and Agriculture, Sacramento, California, USA; f A. A. Kharkevich Institute for Information Transmission Problems, Russian Academy of Sciences, Moscow, Russia; University of Arizona

## Abstract

Complete and draft genome sequences of 12 *Rathayibacter* strains were generated using Oxford Nanopore and Illumina technologies. The genome sizes of these strains are 3.21 to 4.61 Mb, with high G+C content (67.2% to 72.7%) genomic DNA. Genomic data will provide useful baseline information for natural taxonomy and comparative genomics of members of the genus *Rathayibacter*.

## ANNOUNCEMENT

The genus *Rathayibacter* (*Actinobacteria*) comprises eight species with validly published names (1–6). In addition, some putative new species of this genus have been discovered, including “Rathayibacter tanaceti” (7–10). The species R. rathayi, R. iranicus, R. tritici, and R. toxicus are well-known plant pathogens causing a gumming disease of wheat and cereal grasses (4). *R. toxicus* is also responsible for toxicity of annual ryegrass and some other grasses, which often results in poisoning of grazing animals (7, 8). *Rathayibacter* species are transmitted to their host plants by seed gall nematodes of the genus *Anguina* (Anguinidae) (4, 11). Four additional *Rathayibacter* species were found in plant galls induced by the leaf gall nematode Anguina graminis (R. festucae) (3), in a diseased wheatgrass (R. agropyri) (6), and also in plants without any visible symptoms of bacterial diseases or nematode infestation (R. caricis and R. oskolensis) (3, 5).

Novel *Rathayibacter* strains were recovered from Tanacetum vulgare (Asteraceae) infested by the foliar nematode Aphelenchoides fragariae (Aphelenchoididae) and from plants with no visible disease symptoms (Table 1). All strains were isolated as described previously (5) and deposited in the All-Russian Collection of Microorganisms (VKM; http://www.vkm.ru). The universal bacterial primers 27F (5′-AGAGTTTGATCCTGGCTCAG-3′) and 1525R (5′-AAGGAGGTGATCCAGCC-3′) were used for 16S rRNA gene amplification and sequencing. The pairwise similarity between the 16S rRNA gene sequences was determined using TaxonDC (12). The strains showed 97.5% to 99.9% 16S rRNA gene sequence similarities with validly described *Rathayibacter* species.

**TABLE 1:** Statistical information for genome sequences and DDBJ/ENA/GenBank accession numbers

Organism	Plant	Nematode	No. of long reads	*N*_50_ (bp) of long reads	No. of short reads^*a*^	Coverage (×)	No. of contigs	Contig *N*_50_ (bp)	Genome size (Mbp)	G+C content (%)	No. of complete plasmids	No. of proteins	Completeness	SRA accession no.	GenBank accession no.
*Rathayibacter* sp. VKM Ac-2759	*Tanacetum vulgare*	*A. fragariae*	105,881	8,604	10,526,398	442			4.16	71.6	3	3,814	Complete	SRR10912284, SRR10912285	CP047176, CP047177, CP047178, CP047179
*Rathayibacter* sp. VKM Ac-2760	*Tanacetum vulgare*	*A. fragariae*	41,508	4,270	12,240,072	378			4.61	72.1	2	4,107	Complete	SRR10912303, SRR10912304	CP047173, CP047174, CP047175
“*R. tanaceti*” VKM Ac-2761	*Tanacetum vulgare*	*A. fragariae*	70,773	9,437	23,061,818	1,111			3.21	70.7		2,932	Complete	SRR10912305, SRR10912306	CP047186
*Rathayibacter* sp. VKM Ac-2801	Androsace koso-poljanskii	No	52,705	8,511	19,740,286	791			3.63	72.3	1	3,317	Complete	SRR10912288, SRR10912289	CP047183, CP047184
*R. festucae* VKM Ac-2802	*Androsace koso-poljanskii*	No	80,390	4,226	17,945,598	572			4.32	72.4	2	3,871	Complete	SRR10912286, SRR10912287	CP047180, CP047181, CP047182
*Rathayibacter* sp. VKM Ac-2805	Gypsophila altissima	No	175,323	4,603	9,212,982	431			3.6	72.4		3,285	Complete	SRR10912290, SRR10912294	CP047185
*Rathayibacter* sp. VKM Ac-2762	*Limonium* sp.	No	36,401	3,682	7,531,042	302			3.45	72.7		3,151	Complete^*b*^	SRR10912299, SRR10912300	CP047419
*Rathayibacter* sp. VKM Ac-2804	Koeleria macrantha	No	91,359	5,322	9,828,426	374			4.09	72.4		3,686	Complete^*b*^	SRR10912301, SRR10912302	CP047420
R. rathayi VKM Ac-1601^T^	Dactylis glomerata	*Anguina* sp.			9,771,504	401	60	256,770	3.21	69.3		2,983	Draft	SRR10912291	WUCA00000000
R. iranicus VKM Ac-1602^T^	Triticum aestivum	*Anguina tritici*	3,667	4,472	14,405,148	542	62	193,466	3.38	67.2		3,121	Draft	SRR10912292, SRR10912293	WUCB00000000
*Rathayibacter* sp. VKM Ac-2754	*Androsace koso-poljanskii*	No	4,359	3,645	3,293,486	112	24	431,504	3.97	71.6	1	3,660	Draft	SRR10912295, SRR10912296	WUCC00000000
*Rathayibacter* sp. VKM Ac-2803	*Androsace koso-poljanskii*	No	57,177	5,352	22,330,660	753	4	3,988,627	4.29	71.3	2	3,978	Draft	SRR10912297, SRR10912298	WUCD00000000

a 150-bp paired-end reads.

b Chromosome contains one gap.

For DNA extraction, biomass was grown in liquid peptone-yeast medium (13) inoculated with cells from a single colony, followed by cultivation at 28°C for 18 to 20 h on a rotary shaker. Genomic DNA was extracted using a QIAamp DNA minikit (Qiagen, Germany).

DNA libraries were prepared for long-read sequencing using the Nanopore rapid barcoding genomic DNA (gDNA) sequencing kit (catalog number SQK-RBK004; Oxford Nanopore Technologies) according to the manufacturer’s protocol and were sequenced in-house using a MinION device.

DNA libraries of strains VKM Ac-2754, VKM Ac-2759, VKM Ac-2760, VKM Ac-2762, VKM Ac-2804, and VKM Ac-2805 were prepared for short-read sequencing using the Nextera DNA flex library prep kit (Illumina) and Nextera DNA CD indexes (Illumina) according to the manufacturer’s instructions. DNA libraries of strains VKM Ac-1601^T^, VKM Ac-1602^T^, VKM Ac-2761, VKM Ac-2801, VKM Ac-2802, and VKM Ac-2803 were prepared using NEBNext Ultra II FS DNA library prep kit for Illumina (New England BioLabs) following the protocol for use with inputs of ≥100 ng with the following modifications: TruSeq DNA CD indexes (Illumina) were used in place of NEBNext adaptors to eliminate the need for PCR steps. The USER enzyme addition was skipped for this reason, and the volume was adjusted with water to reach the necessary sample volume for size selection steps. No PCR amplification was performed on these libraries. Pooled DNA libraries were sequenced by Novogene Co., Ltd.

Default parameters were used for all software unless otherwise specified. Nanopore basecalling was performed by Guppy basecalling software 2.3.5, available from the Oxford Nanopore Technology (ONT) community website (with the following parameters: --flowcell, FLO-MIN106; --kit, SQK-RBK004), and demultiplexed by Deepbinner 0.2.0 (14) (with parameter --rapid). Adapter sequences from long reads were removed using Porechop 0.2.4 (https://github.com/rrwick/Porechop) with parameter --discard_middle. Adapter sequences and low-quality regions in short reads were cut using Trimmomatic 0.39 (15) with the following parameters: ILLUMINACLIP:adapters.fa:2:30:10; SLIDINGWINDOW:4:15; MINLEN:30, where adapters.fa is NexteraPE-PE.fa or TruSeq3-PE-2.fa depending on the kit used to prepare the library. Hybrid assembly was performed by Unicycler 0.4.8 (16). There was insufficient DNA quantity of VKM Ac-1601^T^ to make a library for Nanopore sequencing; thus, the genome assembly of this organism was performed on short reads only. The quality of assemblies was assessed with QUAST 5.0.2 (17). Assemblies were annotated with the NCBI PGAP (18) and the RAST Web server (19, 20). A phylogenomic tree was inferred by the balanced minimum evolution method using JolyTree (21). Statistical information for the complete and draft genome sequences is given in Table 1. It is worth noting that plasmids were identified in the genome assemblies of *Rathayibacter* strains for the first time.

**FIG 1:**
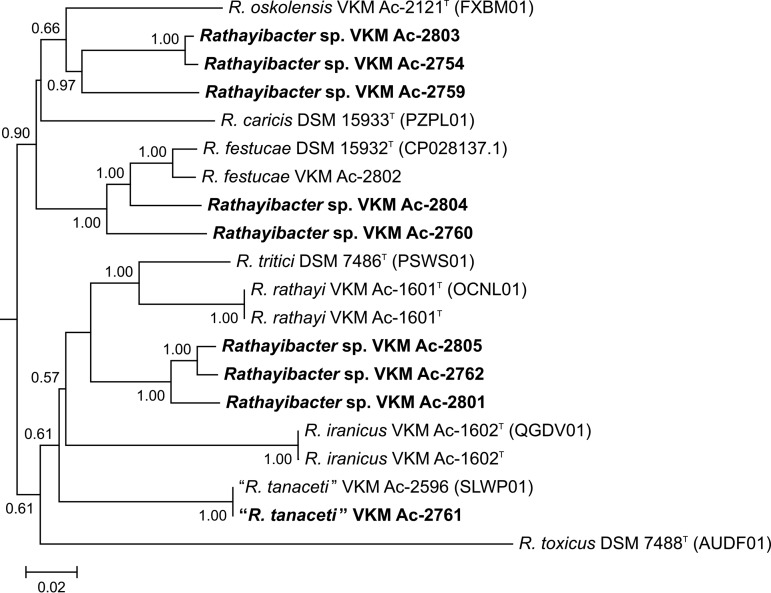
Phylogenomic tree based on 20 bacterial genomes of the genus *Rathayibacter*. The tree is drawn to scale, with branch lengths measured in the estimated number of substitutions per site. Branch support values (rate of elementary quartets) above 0.5 are indicated at the branch points. The following newly isolated strains of seven putative new species are given in bold: (i) VKM Ac-2803 and VKM Ac-2754, (ii) VKM Ac-2759, (iii) VKM Ac-2804, (iv) VKM Ac-2760, (v) VKM Ac-2805 and VKM Ac-2762, (vi) VKM Ac-2801, and (vii) VKM Ac-2761 (the second strain of “*R. tanaceti*”). The genomic sequence of *Clavibacter sepedonicus* ATCC 33113^T^ (GenBank accession numbers AM849034.1 to AM849036.1) served as an outgroup (not shown).

The tree (Fig. 1) shows that 9 of the 10 novel strains cluster separately from the *Rathayibacter* species with validly published names. The calculated average nucleotide identity (ANI) and digital DNA-DNA hybridization (dDDH) values (well below the borderlines for species differentiation [22]; not shown) indicated the presence of seven putative new species among the strains studied. Further comparative phenotypic study and genome-wide analyses of these strains and other members of the genus *Rathayibacter* will result in valid descriptions of the revealed new species and facilitate insight into the molecular mechanisms involved in interactions between plants and bacteria.

### Data availability.

These whole-genome shotgun projects have been deposited in DDBJ/ENA/GenBank under the accession numbers listed in Table 1. The versions reported here are the first versions. The accession numbers of the 16S rRNA gene sequences deposited in DDBJ/ENA/GenBank are MT431563 to MT431574.

## References

[1] Zgurskaya HI, Evtushenko LI, Akimov VN, Kalakoutskii LV 1993 *Rathayibacter* gen. nov., including the species *Rathayibacter rathayi* comb. nov., *Rathayibacter tritici* comb. nov., *Rathayibacter iranicus* comb. nov., and six strains from annual grasses. Int J Syst Bacteriol 43:143–149. doi:10.1099/00207713-43-1-143.

[2] Sasaki J, Chijimatsu M, Suzuki K-I 1998 Taxonomic significance of 2,4-diaminobutyric acid isomers in the cell wall peptidoglycan of actinomycetes and reclassification of *Clavibacter toxicus* as *Rathayibacter toxicus* comb. nov. Int J Syst Bacteriol 48:403–410. doi:10.1099/00207713-48-2-403. 9731278

[3] Dorofeeva LV, Evtushenko LI, Krausova VI, Karpov AV, Subbotin SA, Tiedje JM 2002 *Rathayibacter caricis* sp. nov. and *Rathayibacter festucae* sp. nov., isolated from the phyllosphere of *Carex* sp. and the leaf gall induced by the nematode *Anguina graminis* on *Festuca rubra* L., respectively. Int J Syst Evol Microbiol 52:1917–1923. doi:10.1099/00207713-52-6-1917. 12508848

[4] Evtushenko LI, Dorofeeva LV 2012 Genus XXII. *Rathayibacter* Zgurskaya, Evtushenko, Akimov and Kalakoutskii 1993, 147, p 953–964. In Goodfellow M, Kämpfer P, Busse H-J, Trujillo ME, Suzuki K-I, Ludwig W, Whitman WB (ed), Bergey’s manual of systematic bacteriology, 2nd ed, vol 5 Springer, New York, NY.

[5] Dorofeeva LV, Starodumova IP, Krauzova VI, Prisyazhnaya NV, Vinokurova NG, Lysanskaya VY, Tarlachkov SV, Evtushenko LI 2018 *Rathayibacter oskolensis* sp. nov., a novel actinobacterium from *Androsace koso-poljanskii* Ovcz. (Primulaceae) endemic to Central Russian Upland. Int J Syst Evol Microbiol 68:1442–1447. doi:10.1099/ijsem.0.002681. 29517475

[6] Schroeder BK, Schneider WL, Luster DG, Sechler A, Murray TD 2018 *Rathayibacter agropyri* (non O’Gara 1916) comb. nov., nom. rev., isolated from western wheatgrass (Pascopyrum smithii). Int J Syst Evol Microbiol 68:1519–1525. doi:10.1099/ijsem.0.002708. 29557775

[7] Murray TD, Schroeder BK, Schneider WL, Luster DG, Sechler A, Rogers EE, Subbotin SA 2017 *Rathayibacter toxicus*, other *Rathayibacter* species inducing bacterial head blight of grasses, and the potential for livestock poisonings. Phytopathology 107:804–815. doi:10.1094/PHYTO-02-17-0047-RVW. 28414631

[8] Riley IT, Swart A, Postnikova E, Agarkova I, Vidaver AK, Schaad NW 2004 New association of a toxigenic *Rathayibacter* sp. and *Anguina woodi* in *Ehrhata villosa* var. *villosa* in South Africa. Phytopathology 94:S88.

[9] Starodumova IP, Tarlachkov SV, Prisyazhnaya NV, Dorofeeva LV, Ariskina EV, Chizhov VN, Subbotin SA, Evtushenko LI, Vasilenko OV 2017 Draft genome sequence of *Rathayibacter* sp. strain VKM Ac-2630 isolated from the leaf gall induced by the knapweed nematode *Mesoanguina picridis* on *Acroptilon repens*. Genome Announc 5:e00650-17. doi:10.1128/genomeA.00650-17. 28751392 PMC5532830

[10] Vasilenko OV, Starodumova IP, Tarlachkov SV, Dorofeeva LV, Avtukh AN, Evtushenko LI 2016 Draft genome sequence of “*Rathayibacter tanaceti*” strain VKM Ac-2596 from *Tanacetum vulgare* infested by a foliar nematode. Genome Announc 4:e00512-16. doi:10.1128/genomeA.00512-16. 27313291 PMC4911470

[11] Riley IT, McKay AC 1990 Specificity of the adhesion of some plant pathogenic microorganisms to the cuticle of nematodes in the genus *Anguina* (Nematoda*: anguinidae*). Nematol 36:90–103. doi:10.1163/002925990X00068.

[12] Tarlachkov SV, Starodumova IP 2017 TaxonDC: calculating the similarity value of the 16S rRNA gene sequences of prokaryotes or ITS regions of fungi. J Bioinform Genom 3:1–4. doi:10.18454/jbg.2017.3.5.1.

[13] Naumova IB, Kuznetsov VD, Kudrina KS, Bezzubenkova AP 1980 The occurrence of teichoic acids in Streptomycetes. Arch Microbiol 126:71–75. doi:10.1007/BF00421893. 7396640

[14] Wick RR, Judd LM, Holt KE 2018 Deepbinner: demultiplexing barcoded Oxford Nanopore reads with deep convolutional neural networks. PLoS Comput Biol 14:e1006583. doi:10.1371/journal.pcbi.1006583. 30458005 PMC6245502

[15] Bolger AM, Lohse M, Usadel B 2014 Trimmomatic: a flexible trimmer for Illumina sequence data. Bioinformatics 30:2114–2120. doi:10.1093/bioinformatics/btu170. 24695404 PMC4103590

[16] Wick RR, Judd LM, Gorrie CL, Holt KE 2017 Unicycler: resolving bacterial genome assemblies from short and long sequencing reads. PLoS Comput Biol 13:e1005595. doi:10.1371/journal.pcbi.1005595. 28594827 PMC5481147

[17] Gurevich A, Saveliev V, Vyahhi N, Tesler G 2013 QUAST: quality assessment tool for genome assemblies. Bioinformatics 29:1072–1075. doi:10.1093/bioinformatics/btt086. 23422339 PMC3624806

[18] Tatusova T, DiCuccio M, Badretdin A, Chetvernin V, Nawrocki EP, Zaslavsky L, Lomsadze A, Pruitt KD, Borodovsky M, Ostell J 2016 NCBI Prokaryotic Genome Annotation Pipeline. Nucleic Acids Res 44:6614–6624. doi:10.1093/nar/gkw569. 27342282 PMC5001611

[19] Aziz RK, Bartels D, Best AA, DeJongh M, Disz T, Edwards RA, Formsma K, Gerdes S, Glass EM, Kubal M, Meyer F, Olsen GJ, Olson R, Osterman AL, Overbeek RA, McNeil LK, Paarmann D, Paczian T, Parrello B, Pusch GD, Reich C, Stevens R, Vassieva O, Vonstein V, Wilke A, Zagnitko O 2008 The RAST server: Rapid Annotations using Subsystems Technology. BMC Genomics 9:75. doi:10.1186/1471-2164-9-75. 18261238 PMC2265698

[20] Overbeek R, Olson R, Pusch GD, Olsen GJ, Davis JJ, Disz T, Edwards RA, Gerdes S, Parrello B, Shukla M, Vonstein V, Wattam AR, Xia F, Stevens R 2014 The SEED and the Rapid Annotation of microbial genomes using Subsystems Technology (RAST). Nucleic Acids Res 42:D206–D214. doi:10.1093/nar/gkt1226. 24293654 PMC3965101

[21] Criscuolo A 2019 A fast alignment-free bioinformatics procedure to infer accurate distance-based phylogenetic trees from genome assemblies. Res Ideas Outcomes 5:e36178. doi:10.3897/rio.5.e36178.

[22] Chun J, Oren A, Ventosa A, Christensen H, Arahal DR, da Costa MS, Rooney AP, Yi H, Xu XW, De Meyer S, Trujillo ME 2018 Proposed minimal standards for the use of genome data for the taxonomy of prokaryotes. Int J Syst Evol Microbiol 68:461–466. doi:10.1099/ijsem.0.002516. 29292687

